# Stool Culture for Diagnosis of Pulmonary Tuberculosis in Children

**DOI:** 10.1128/JCM.00801-17

**Published:** 2017-11-27

**Authors:** Elisabetta Walters, Anne-Marie Demers, Marieke M. van der Zalm, Andrew Whitelaw, Megan Palmer, Corné Bosch, Heather R. Draper, Robert P. Gie, Anneke C. Hesseling

**Affiliations:** a Desmond Tutu TB Centre, Faculty of Medicine and Health Sciences, Stellenbosch University, Cape Town, South Africa; b Division of Medical Microbiology, National Health Laboratory Service, Tygerberg Hospital, Cape Town, South Africa; Johns Hopkins University School of Medicine

**Keywords:** childhood tuberculosis, stool culture, diagnosis

## Abstract

Bacteriological confirmation of Mycobacterium tuberculosis is achieved in the minority of young children with tuberculosis (TB), since specimen collection is resource intensive and respiratory secretions are mostly paucibacillary, leading to limited sensitivity of available diagnostic tests. Although molecular tests are increasingly available globally, mycobacterial culture remains the gold standard for diagnosis and determination of drug susceptibility and is more sensitive than molecular methods for paucibacillary TB. We evaluated stool culture as an alternative to respiratory specimens for the diagnosis of suspected intrathoracic TB in a subgroup of 188 children (median age, 14.4 months; 15.4% HIV infected) enrolled in a TB diagnostic study at two local hospitals in Cape Town, South Africa. One stool culture was compared to overall bacteriological confirmation by stool Xpert and by Xpert and culture of multiple respiratory specimens. After decontamination/digestion with NALC (*N*-acetyl-l-cysteine)-NaOH (1.25%), concentrated fluorescent smear microscopy, Xpert MTB/RIF, and liquid culture were completed for all specimens. Culture contamination of stool specimens was high at 41.5%. Seven of 90 (7.8%) children initiating TB treatment were stool culture positive for M. tuberculosis. Excluding contaminated cultures, the sensitivity of stool culture versus confirmed TB was 6/25 (24.0%; 95% confidence interval [CI] = 9.4 to 45.1%). In addition, stool culture detected TB in 1/93 (1.1%) children with “unconfirmed TB.” Testing the same stool by Xpert increased sensitivity to 33.3% (95% CI = 18.0 to 51.8%). In conclusion, stool culture had low sensitivity for M. tuberculosis detection in children with intrathoracic TB. Reducing culture contamination through improved laboratory protocols may enable more reliable estimates of its diagnostic utility.

## INTRODUCTION

Although rapid molecular methods are increasingly being adopted globally for the diagnosis of tuberculosis (TB) (1), culture-based methods remain the reference (gold) standard for the diagnosis of TB and for drug susceptibility testing (DST) (1). In paucibacillary forms of TB, including most forms of TB in children and sputum-scarce or smear-negative adults with HIV-associated pulmonary TB (PTB), detection by culture is considerably superior to molecular assays. For example, the Xpert MTB/RIF assay (Xpert; Cepheid, Sunnydale, CA) has 62 to 66% sensitivity compared to culture for the diagnosis of pediatric PTB (2), and 68% for smear-negative PTB in adults (3). However, even culture in these patient groups confirms <50% of cases (4–6), partly due to low bacterial burden and the difficulty in obtaining high-quality sputum specimens (6, 7).

TB in children, although under-reported, contributes at least 10% of the disease burden globally (1), and up to 21% (8) of the total TB case load in high-burden TB settings. Children generally have good TB treatment outcomes given timely diagnosis and treatment; however, immunological immaturity, especially in young and HIV-infected children, favors rapid progression of TB disease if the diagnosis is missed or delayed (9, 10).

In addition to the importance of confirming a diagnosis, especially in children from high-risk groups, the increasing incidence of drug-resistant TB globally (1) calls for greater efforts to pursue bacteriological confirmation in all patients at risk of drug resistance, including children, in order to treat all patients effectively and to prevent the emergence and transmission of resistant M. tuberculosis strains (11, 12).

Alternative methods to collect respiratory specimens, such as sputum induction, gastric aspiration, and bronchoalveolar lavage from young children, are resource intensive and relatively invasive. When collected, these specimens typically have a low mycobacterial burden, resulting in modest detection by current available tests, including mycobacterial culture and molecular assays (2, 5, 13). Collecting multiple specimens improves detection yield (14–16) but is costly and typically has to occur over consecutive days, which limits its feasibility.

Stool is easily collected from children and can be used for the detection of M. tuberculosis present in swallowed sputum, using both culture and molecular methods (17–26). We have recently shown that stool Xpert detects approximately one in four children with radiologically severe PTB (25). Although Xpert gives rapid results, including rifampin resistance, current diagnostic algorithms still require a cultured isolate for further DST to isoniazid and other drugs. We evaluated stool culture as a noninvasive strategy for the diagnosis of intrathoracic (pulmonary) TB in a subset of children with suspected PTB, whose stool Xpert results were reported previously (25).

## RESULTS

**FIG 1:**
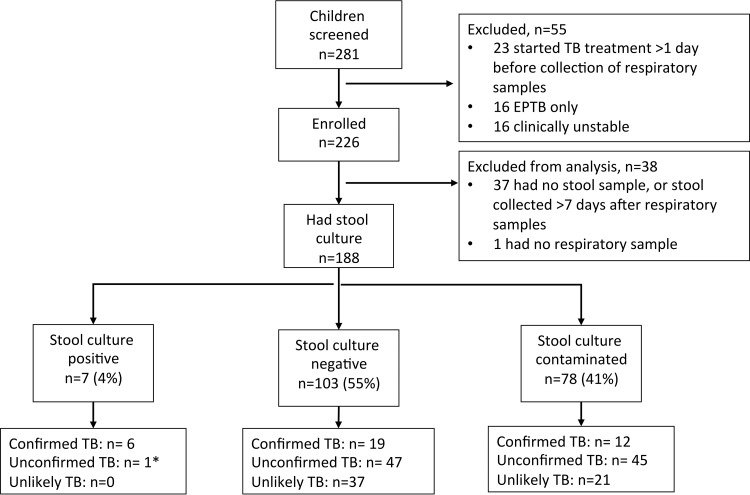
STARD cohort flow diagram, illustrating stool culture results by consensus case definition (27). EPTB, extrapulmonary tuberculosis. *, One child was positive on stool culture only; all respiratory cultures and Xpert and stool Xpert were negative.

Cohort recruitment and characteristics are summarized in Fig. 1 and Table 1. Overall, 188 children were included in the study. Thirty-seven (19.7%) children were classified as “confirmed TB” (excluding stool culture results as the index test), 93 (49.5%) were classified as “unconfirmed TB,” and 58 (30.9%) were classified as “unlikely TB” according to international consensus clinical case definitions for intrathoracic TB in children (27). Overall, 90 (47.9%) children were treated for TB by the clinical care team. Twenty-six of 90 (28.9%) children who initiated antituberculosis therapy had stool specimens collected after treatment initiation (median, 2 days; interquartile range, 1 to 4 days).

**TABLE 1:** Cohort characteristics overall and grouped by international consensus diagnostic category (27) in children presenting with suspected tuberculosis (*n* = 188)

Characteristic	Diagnosis^*a*^			
	All children	Children with confirmed TB^*b*^	Children with unconfirmed TB	Children with unlikely TB
Total	188 (100)	37 (19.7)	93 (49.5)	58 (30.9)
Age (mo)	14.4	17.5	15.5	12.5
Median (IQR)	7.2–25.6	8.9–28.4	9.1–26.1	5.6–20.2
Male	95 (50.0)	15 (40.5)	48 (51.6)	32 (55.2)
HIV infected	29 (15.4)	2 (5.4)	19 (20.4)	8 (13.8)
On ART at presentation	9 (31.0)	0 (0)	8 (42.1)	1 (12.5)
WAZ<-2	97 (51.6)	19 (51.4)	59 (63.4)	19 (32.8)
With evidence of BCG immunization	182 (96.8)	32 (86.5)	93 (100)	57 (98.3)
≥1 well-defined TB symptom(s)^*c*^	149 (79.3)	33 (89.2)	81 (87.1)	35 (60.3)
TST positive	48 (29.4)	23 (74.2)	21 (25.6)	4 (8.0)
*n*	163	31	82	50
Exposure to identified TB source case	105 (55.9)	25 (67.6)	71 (76.3)	9 (15.5)
CXR suggestive of TB	58 (31.9)	26 (74.3)	28 (30.1)	4 (7.4)
*n*	182	35	93	54
Treated for TB	90 (47.9)	37 (100)	38 (40.9)	15 (25.9)

a Values are expressed as number (%) unless otherwise noted in column 1. IQR, interquartile range; ART, antiretroviral therapy; WAZ, weight-for-age Z-score according to UK growth charts 1990 (44); BCG, bacillus Calmette-Guérin; TST, tuberculin skin test; CXR, chest radiograph.

b This value includes all children with positive Xpert or culture of M. tuberculosis from respiratory specimens or a positive Xpert result for stool. Two children were confirmed only on stool Xpert. One child whose only M. tuberculosis-positive test was stool culture is classified as “unconfirmed TB,” since stool culture was the index test.

c As reported previously (45).

Of the 37 children with bacteriologically confirmed TB, 28 were confirmed on respiratory specimen culture and 9 were confirmed by Xpert only (7 on respiratory specimens and two on stool; all 9 were culture negative). Stool culture was positive in 6/37 (16.2%) children with “confirmed TB,” in 1/93 (1.1%) children with “unconfirmed TB” and in none of the children with “unlikely TB.”

In order to present a fair comparison between stool and respiratory specimens, we also compared stool culture to culture of respiratory specimens in children who had stool and respiratory specimens collected on the same day (*n* = 153). Stool culture sensitivity was 33.3% (95% confidence interval [CI] = 11.8 to 61.6%), with other measures of diagnostic accuracy being similar to the comparison between stool and 2 gastric aspirate (GA) or 2 sputum (SPT) specimens. The diagnostic accuracy analyses for stool culture are shown in Table 2 and detailed microbiology of stool specimens in Table 3.

**TABLE 2:** Diagnostic accuracy of stool culture compared to defined reference standards^*a*^

Parameter	Comparison			
	SC vs culture or Xpert^*b*^	SC vs clinical decision to treat^*c*^	SC vs culture of GA/SPT^*d*^	SC vs culture of respiratory specimens^*e*^
Stool culture result (no. of samples [+/–/total])				
Stool culture (+)	6/1/7	7/0/7	5/2/7	5/1/6
Stool culture (–)	15/88/103	46/57/103	10/91/101	10/73/83
Totals	21/89/110	53/57/110	15/93/108	15/74/89
% sensitivity or specificity (95% CI)				
Sensitivity	28.6 (11.3, 52.2)	13.2 (5.5, 25.3)	33.3 (11.8, 61.6)	33.3 (11.8, 61.6)
Specificity	98.9 (93.9, 100.0)	100.0 (93.7, 100.0)	97.8 (92.4, 99.7)	98.6 (92.7, 100.0)
PPV or NPV (95% CI)				
PPV	85.7 (42.1, 99.6)	100.0 (59.0, 100.0)	71.4 (29.0, 96.3)	83.3 (35.9, 99.6)
NPV	85.4 (77.1, 91.6)	55.3 (45.2, 65.1)	90.1 (82.5, 95.1)	88.0 (79.0–94.1)

a GA, gastric aspirate; SPT, sputum; IS, induced sputum; (+), positive; (–), negative; CI, confidence interval; PPV, positive predictive value; NPV, negative predictive value.

b That is, stool culture compared to culture or Xpert of up to 2 GA/SPT and 2 IS specimens; *n* = 110 (78 children with contaminated stool culture were excluded).

c That is, stool culture compared to clinical decision to treat; *n* = 110 (78 children with contaminated stool culture were excluded).

d That is, stool culture compared to culture of 2 GA/SPT specimens; *n* = 108 (78 children with contaminated stool culture and 2 with contaminated GA cultures were excluded).

e That is, stool culture compared to culture of respiratory specimens collected on the same day; *n* = 89 (64 children with contaminated stool culture and 2 with contaminated respiratory cultures were excluded).

**TABLE 3:** Stool microbiology results grouped by culture, Xpert MTB/RIF, and smear results^*a*^

Culture	TTP (days)	Xpert	Xpert semiquantitative	Smear	Smear grade	No. of stool specimens^*b*^ (*n* = 188)
Pos MTB	9	Det	VL	Pos	1+	1
	16	Det	L	Neg		1
	25	Det	L	Neg		1
	26	Det	L	Neg		1
	19	Neg		Neg		1
	22	Neg		Neg		1
	12	E/I/NR		Pos	1+	1
Neg		Det	M	Pos	3+	1
		Det	VL	Pos	2+	1
		Det	L	Pos	Scanty	1
		Det	VL	Neg		1
		Det	VL	Neg		1
		Det	L	Neg		1
		Neg		Neg		85
		E/I/NR		Neg		12
Contaminated		Det	VL	Neg		1
		Neg		Neg		69
		E/I/NR		Neg		8

a TTP, time to positivity; Pos, positive; Det, M. tuberculosis detected; VL, very low; L, low; Neg, negative; E/I/NR, error, invalid, or no result; M, medium.

b One stool specimen per participant (*n* = 188 participants).

If contaminated stool cultures are excluded, there was no difference in culture positivity for stool specimens collected before (4/35; 11.4%) or after (3/18; 16.7%) initiation of TB treatment (*P* = 0.667). Of the children with positive respiratory cultures (*n* = 28), 21 (75%) had stool collected within 1 day of the first culture-positive respiratory specimen: 12/21 (57.1%) on the same day or 1 day before the respiratory specimen and 9/21 (42.9%) 1 day after the respiratory specimen. Stool cultures were positive in 6 of these 21 (28.6%) children; 10/21 (47.6%) stool cultures were negative, and 5/21 (23.8%) were contaminated. In comparison, none of the 7 stools collected >1 day after the first culture-positive respiratory specimen were culture positive.

The 7 isolates from positive stool cultures were all drug susceptible. Drug-resistant TB was detected from respiratory specimens in 4/37 (10.8%) children with confirmed TB: 2 were at least isoniazid monoresistant, and 2 were multidrug resistant (MDR), with susceptibility to ofloxacin and amikacin confirmed on phenotypic DST. Stool culture was negative in all 4, despite stool being collected before treatment in all cases.

Two of the seven (28.6%) culture-positive and 3/103 (2.9%) culture-negative stool specimens were smear positive (Table 3). The three culture-negative, smear-positive stool specimens were all collected before starting TB treatment, and all were processed between 12 and 72 h after collection. All smear-positive stool samples originated from children who had confirmed TB: respiratory specimens from these children were all smear, Xpert, and culture positive. No nontuberculous mycobacteria were isolated from stool.

Culture was contaminated in 78/188 (41.5%) stool versus 31/419 (7.4%) GA, 24/425 (5.7%) induced sputum (IS), and 0/6 (0.0%) SPT specimens. Although not subjected to microbiological identification, contaminating organisms were compatible with fungal and bacterial overgrowth.

### Incremental value of Xpert on stool for M. tuberculosis detection.

Of the seven stool specimens with positive cultures, the Xpert result on the same specimens was positive in four (57.1%). However, the incremental value of testing all stool specimens with Xpert as well was 100%, since Xpert detected M. tuberculosis in an additional seven stool specimens, six of which were stool culture negative and one had a contaminated culture (Table 3). The sensitivity/specificity of combined stool culture and Xpert versus confirmed TB based on respiratory specimens alone was 33.3% (95% CI = 18.0 to 51.8%)/98.0% (95% CI = 94.2 to 99.6%), respectively, if including participants with either a valid stool Xpert or stool culture result (*n* = 180). Eight children who had both stool Xpert invalid and stool culture contaminated were excluded from this calculation. The reduced specificity is due to three children who were positive on stool Xpert or stool culture alone, but negative on all respiratory specimens. None of the children with MDR-TB were stool Xpert positive.

### Spectrum of TB disease in children with positive stool culture.

Six of the seven children with positive stool cultures showed severe disease on chest radiographs (CXR) (28), including four with cavities. One child had a normal CXR, and all cultures except stool were negative. The child was started on antituberculosis therapy clinically before stool culture results were available, based on suggestive symptoms, a positive tuberculin skin test (TST), and significant TB exposure history (mother with TB), and responded well to TB treatment. None of the seven had extrathoracic TB.

### Stool culture in abdominal TB.

Of seven children with a clinical/sonographic diagnosis of abdominal TB, four had negative and three had contaminated stool cultures. For one child, M. tuberculosis was detected by Xpert in a stool sample.

## DISCUSSION

The ability to culture M. tuberculosis from an appropriate clinical specimen allows for characterization of the mycobacterial isolate, including genotyping and DST, and remains a critical part of clinical management of TB in children and adults. We have already shown that in children with severe intrathoracic TB, stool Xpert can provide a rapid confirmation in a substantial proportion of children and can directly inform clinical care (25). Since culture is more sensitive than Xpert, especially in paucibacillary TB (2, 29), we evaluated the diagnostic utility of stool culture in a subgroup of children whose stool Xpert results we had previously reported on. However, stool culture was discontinued early due to the high contamination rate relative to its poor diagnostic yield. Stool culture did not confirm any of the children with drug-resistant TB and was positive only in 4% of children overall, mostly children with severe manifestations of PTB.

We found that adding Xpert testing of the same stool specimens increased M. tuberculosis detection by 100%, since seven stool specimens were Xpert positive but culture negative or contaminated, thereby adding seven additional confirmed diagnoses to the seven confirmed by stool culture. Although Xpert testing had better sensitivity than culture for stool, Xpert only allows partial DST: combining the two testing methods could improve the sensitivity of stool testing, while simultaneously enabling at least a portion of specimens to undergo full DST if clinically relevant. In our cohort, none of the children with MDR-TB were detected using either stool culture or stool Xpert. This may be due to chance since the numbers were small. Studies enrolling children with suspected drug-resistant TB would be best placed to opportunistically evaluate the diagnostic utility of stool culture and Xpert in this patient population.

The sensitivity of stool culture compared to mycobacteriological confirmation using respiratory specimens was 24%, excluding contaminated cultures. If these are included as “not positive,” the sensitivity was even lower at 16.2%. This is because some of children excluded based on contaminated stool culture in the first calculation had confirmed TB, so the relative proportion of stool culture-positive children was higher (data not shown). Allowing stool to be collected after TB treatment initiation (up to 7 days) is a limitation of this study and may have contributed to lower bacillary numbers in stool. In contrast, according to protocol entry criteria, respiratory specimens were mostly collected pretreatment. Although our data suggest that pretreatment collection of stool was not associated with a higher proportion of positive stool cultures, this analysis was not adequately powered. Notably, all culture-positive stools came from children whose stool was collected no more than 1 day after the first culture-positive respiratory specimen. This is probably a function of a correlation between stool and sputum mycobacterial loads. Collection of stool a few days after respiratory specimens may result in lower detection from stool due to a variety of factors, such as treatment with antibiotics and antituberculosis drugs and reduced sputum production leading to less sputum being swallowed.

An additional limitation of our study, which may have negatively biased the stool culture results, is that a single stool specimen was compared to multiple respiratory specimens. We could not find any published studies evaluating the incremental diagnostic yield of additional stool specimens, but it is plausible that, similarly to respiratory specimens, increasing the number of specimens collected could have an additive effect. Although mindful of these limitations, we opted for a pragmatic approach to stool collection, since we were evaluating the potential of stool testing as a feasible strategy for resource-limited settings. Applying excessively strict conditions for method and timing of collection would severely limit feasibility and is less likely to be applicable on the field. Although the cost of a second culture may be prohibitive in many settings, the value of a second stool culture should be studied and considered for settings where this may be an option.

The sensitivity of stool culture in our study, although low, is higher than that reported in the other pediatric studies on stool culture for TB diagnosis by Donald and Oberhelman, where the sensitivity was 15 to 20% compared to culture of two gastric aspirates (20, 21, 30). Our study applied relatively narrow entry criteria, which resulted in almost 50% of the cohort initiating TB treatment, and in a considerable proportion having bacteriologically confirmed TB (42%). In absolute numbers, our study had almost double the number of bacteriologically confirmed cases compared to the other three studies, likely indicating a more severe spectrum of TB disease in our hospital-based cohort. The high prevalence of disease resulted in a high positive predictive value of stool culture (85%), which may not be generalizable to all settings and will be highly dependent on the selection criteria for investigation and on the expected prevalence and severity of TB disease.

It is also difficult to compare our results to the other pediatric studies, as different protocols for stool preparation and different culture methods were used. Oberhelman et al. used a small initial stool mass (0.1 g) diluted in 6 ml of phosphate-buffered saline (PBS) (20, 30), whereas Donald et al. combined two stool specimens (final mass not specified) (21) and followed the method published by Allen (31). All three studies used 1% final concentration sodium hydroxide (NaOH) for decontamination, followed by centrifugation. For culture, Donald et al. used the Bactec radiometric culture, while Oberhelman et al. used both Lowenstein-Jensen and microscopic observation drug susceptibility methods. We used a Mycobacteria Growth Indicator Tube (MGIT; Becton Dickinson, Sparks, MD) culture with PANTA, which is more sensitive than solid culture (32) and is the method used by the South African National Health Laboratory Service. However, despite the addition of antibiotics, MGIT culture is more prone to contamination by commensal microorganisms (32). The abundant microflora which constitutes stool grows rapidly in culture and prevents the identification of the slower-growing M. tuberculosis bacilli. The other published pediatric stool culture studies do not report on contamination rates, but studies in adults using MGIT report 14 to 38% contaminated cultures (23, 33, 34). Earlier reports using nonselective culture media on stool samples resulted in excessive contamination, for the detection of both M. tuberculosis (35) and Mycobacterium avium complex (MAC) (36, 37), leading to early discontinuation of those protocols. More recently, liquid culture has become widely available and is known to result in higher contamination for sputum and nonsputum samples than solid culture media (32).

Culture contamination was the main reason for discontinuing stool culture in our study. Despite instructing caregivers to keep stool specimens refrigerated and allowing for a maximum 72 h from collection to processing, we did not collect data on the site of collection (home versus hospital): it is possible that ideal conditions were not maintained for stool specimens collected at home, and that this contributed to high contamination rates. Various techniques to reduce stool culture contamination in the laboratory have been evaluated. Allen tested different decontaminating agents and concluded that NaOH was superior to sulfuric acid and alkali precipitation for recovery of M. tuberculosis and decontamination (31). In a separate similar study, NaOH also resulted in higher yield and comparable contamination rates compared to Portaels solution and benzalkonium chloride-1-hexadecylpyridinium chloride (35). El Khechine et al. replaced NaOH decontamination with 0.25% chlorhexidine in their laboratory handling of stool samples (19, 38), citing unpublished data of improved recovery versus contamination compared to NaOH (19). Chlorhexidine is inactive against mycobacteria and may increase the recovery of M. tuberculosis (39, 40). The sensitivity of stool culture in the study by El Khechine is the highest reported for the diagnosis of PTB at 54% (19).

Allen also tried to reduce stool culture contamination by diluting samples after the 1% NaOH digestion/decontamination procedure, before inoculation into culture medium (31). Dilutions of 1:10 substantially reduced contamination without affecting M. tuberculosis yield.

Other stool decontamination methods have been evaluated for the recovery of MAC, including the use of oxalic acid (which resulted in similar contamination rates but improved MAC detection) (36) and testing different concentrations of and time exposure to NaOH (37). Although certain protocols could achieve improved detection, the effect on contamination rates was more variable. Importantly, the pathophysiology of MAC disease in AIDS patients, where disease may be primarily abdominal and rapidly disseminates, may explain the higher sensitivity of stool culture for MAC compared to M. tuberculosis in patients with suspected PTB.

Pediatric stool culture studies for M. tuberculosis detection have not evaluated higher NaOH concentrations and longer exposure times for sample decontamination, nor the effect of sample dilution. However, it is plausible that these modifications may disproportionately affect mycobacterial recovery compared to reduction of bacterial and fungal overgrowth on the already paucibacillary specimens typically collected from children with PTB.

### Conclusions.

Although stool can easily be collected by caregivers and untrained health care workers, stool sample preparation and processing for culture are relatively complex and laboratory protocols have yet to be optimized. Given the available evidence, stool culture for TB diagnosis cannot currently be recommended to replace culture and Xpert of respiratory samples for the diagnosis of intrathoracic TB in children. Culture remains an expensive technique, and the high percentage of nonevaluable results from contamination using standard protocols paired with limited diagnostic sensitivity does not currently justify its routine use.

More work is needed before stool culture can be promoted as a feasible diagnostic strategy for resource-limited settings. Given the limited options for confirming TB in children from high-burden settings, stool culture may still have a role in TB diagnosis as an adjunctive diagnostic measure or in clinical situations where confirmation of TB disease and DST results is critical, but laboratory research should be prioritized over clinical evaluations. Specifically, promising laboratory protocols that have shown better sensitivity and low contamination rates, such as those using chlorhexidine, should be systematically evaluated and compared to current protocols. Optimized laboratory protocols could then be applied to targeted high-risk pediatric populations such as children at risk of MDR-TB, those with HIV infection and those with severe forms of intrathoracic TB, where diagnosis is most critical. This approach would ultimately inform whether stool culture has a place in resource-limited settings with laboratory capacity but inadequate resources for sputum collection in children.

## MATERIALS AND METHODS

Cohort eligibility, enrollment, and investigation have been previously described (25). In brief, following caregiver written consent, children <13 years of age who presented to two referral hospitals in Cape Town, South Africa, with suspected intrathoracic TB (PTB inclusive of pleural effusion and hilar adenopathy) were consecutively enrolled from April 2012 to June 2014. Eligibility was based on ≥1 of the following symptoms: (i) cough lasting ≥2 weeks, (ii) unexplained fever for ≥1 week, or (iii) poor growth or weight loss over the preceding 3 months. We also included children with any cough duration, if ≥1 of the following characteristics were present: (i) exposure to an identified TB source case in the preceding 12 months, (ii) positive TST if previously negative or unknown, or (iii) a CXR suggestive of TB as assessed by the study clinician. Infants <3 months of age with pneumonia unresponsive to appropriate antimicrobials or with unexplained and unresponsive sepsis syndrome were also eligible. We excluded children who had received >1 dose of antituberculosis therapy (excluding isoniazid preventive therapy) before respiratory specimen collection, who had an established alternative diagnosis at screening, or who were unable to commit to follow up. Children with both extrapulmonary and intrathoracic manifestations of TB were eligible.

Investigations included TST (Mantoux, two tuberculin units of PPD RT-23; Statens Serum Institute, Copenhagen, Denmark), HIV testing, and CXR, evaluated by two independent experts. TST results were read 48 to 72 h after placement and were considered positive if the wheal measured ≥10 mm if HIV negative and Mycobacterium bovis bacillus Calmette-Guérin (BCG) vaccinated, ≥5 mm if HIV positive, or not BCG vaccinated. Evidence of BCG vaccination was determined by written record in the immunization card or evidence of BCG scar in the right deltoid area. HIV testing was by HIV DNA PCR in children <18 months of age, and by HIV antibody testing (using enzyme-linked immunosorbent assay [ELISA]) if ≥18 months.

Treatment decisions were made by the attending clinicians based on clinical, epidemiological, and bacteriological findings, including the results of all respiratory and stool specimens collected by the study team. For research purposes, participants were classified using the revised clinical case definitions of intrathoracic TB in children (27), into the categories “confirmed TB” (M. tuberculosis confirmed by culture or Xpert on at least one respiratory specimen or on stool Xpert), “unconfirmed TB” (no bacteriological confirmation of M. tuberculosis and a minimum two of well-defined TB symptoms; proof of TB infection; TB exposure in the past 12 months; CXR suggestive of TB; and favorable response to anti-tuberculosis treatment at 2 months), and “unlikely TB” (no bacteriological confirmation of M. tuberculosis and the criteria for “unconfirmed TB” not met). The classification was determined retrospectively at the 2-month follow-up, when culture results from enrollment were available and treatment response was assessed. Disease severity was determined using a pragmatic modification of a published classification (28), wherein radiologically severe TB cases demonstrated any of the following: (i) complications from typical radiological manifestations of TB (e.g., cavities, expansile pneumonia, and nodal airway obstruction), (ii) bilateral parenchymal involvement, (iii) overall parenchymal involvement more extensive than the total area of the right upper lobe, or (iv) disseminated (miliary) TB. For CXR not typical of TB, criteria 2 and 3 were used to define severe disease.

### Specimen collection, transport, and laboratory methods.

At enrollment, on each of two consecutive days, we collected one specimen of two different types (four specimens in all). Study protocol required two early morning GA samples from young children (<5 years of age) unable to expectorate or two fasting SPT in older children, as well as two IS samples with or without nasopharyngeal suctioning. Standard procedures were followed (25). Some children had additional respiratory specimens collected as clinically relevant. One stool specimen per child was collected within 7 days of enrollment. Caregivers were given verbal and written instructions on how to collect stool. For young children in diapers, a urine bag placed onto the perineum prevented urine from mixing with stool. Formed stool was collected directly from the diaper using a scoop attached to the lid of the stool collection receptacle. For liquid stool, the diaper was fitted inside out or plastic waterproof briefs were fitted under the child's diaper in direct contact with the skin: as soon as stool was passed, the liquid stool was poured or scooped into the stool receptacle. Children who were toilet trained were each given a clean potty into which to pass stool, or they could pass stool onto a sheet of plastic cling film fitted onto a conventional toilet seat. Stool receptacles were premarked to indicate the amount of stool needed (six scoops, equivalent to 2 to 3 g).

Specimens collected in hospital were kept refrigerated and transported to the laboratory in a cool box within 4 h of collection. For stool collected at home after discharge from hospital, caregivers were instructed to keep the specimens in a fridge. The study team was notified by phone when the stool sample was ready, and each specimen was collected from the participant's home. Stool specimens were stored refrigerated for maximum 72 h from collection to processing.

Respiratory specimens were processed at the National Health Laboratory Service Microbiology Laboratory at Tygerberg Hospital. For digestion/decontamination, *N*-acetyl-l-cysteine (NALC)-NaOH was used (final NaOH concentration, 1.25%), before concentrated fluorescent Auramine-O smear microscopy (41), Xpert MTB/RIF, and liquid MGIT culture.

Stool specimens were homogenized with 20 ml of PBS (pH 6.8) by vortexing: 5 ml of the stool-PBS mixture was then processed with NALC-NaOH (1.25%). Similarly to respiratory specimens, after concentration at 3,000 × *g* for 20 min, the pellet was resuspended in 2 ml of PBS: a drop was used for fluorescent Auramine-O smear microscopy (41), 0.5 ml was used for MGIT culture (32), and 1.0 ml was mixed with 2.0 ml of Xpert sample reagent and loaded into the GeneXpert instrument (software v4.4a) according to manufacturer's instructions.

Smears were graded according to the WHO/International Union Against Tuberculosis and Lung Disease system (42). Cultures were incubated for up to 42 days. If no growth was observed, cultures were declared negative. For positive cultures, the time to positivity in days was noted, and a Ziehl-Neelsen (ZN) stain was performed on the culture. If the culture was ZN positive, mycobacterial identification and drug susceptibility for isoniazid and rifampin were completed using MTBDR*Plus* (Hain Life Science, Nehren, Germany). Rifampin-resistant strains underwent phenotypic DST for ofloxacin and amikacin using the agar proportion method. If growth of bacteria/fungi was observed on blood agar plates and/or non-acid-fast bacteria were seen on the ZN smear, the MGIT culture was considered contaminated. Contaminated cultures from respiratory specimens were redecontaminated and reincubated once. Contaminated stool cultures were not further decontaminated as local laboratory experience was that redecontamination was rarely successful. Contaminating organisms were not identified. The laboratory technician who handled the stool cultures was not blind to other microbiology results.

### Statistical analysis.

The primary objective was to evaluate the sensitivity, specificity, and predictive values of the stool culture for the diagnosis of intrathoracic TB in children, compared to (i) confirmed TB, as defined above, and (ii) a clinical decision to treat. In secondary analyses, we compared the diagnostic utility of stool culture to the culture of (i) two GA or two SPT specimens, the reference standard used in similar published studies, and (ii) respiratory specimens collected on the same day as stool. We also evaluated the incremental diagnostic value of Xpert testing of the same stool specimen, and the combined sensitivity of stool Xpert and culture versus confirmed TB from respiratory specimens.

All analyses of diagnostic accuracy were conducted per patient (not per specimen). Children were included in analysis if they had a minimum of one stool and one respiratory specimen collected and if stool was collected within 7 days of the respiratory specimens. Contaminated cultures and invalid/error Xpert results were considered nonevaluable and were not repeated. For diagnostic accuracy calculations, participants were excluded if stool culture was contaminated or if all the results of the respiratory specimens were nonevaluable.

Clinical and demographic characteristics were summarized by clinical case definitions using means and standard deviations if normally distributed and with medians and interquartile ranges if not normally distributed. The chi-squared test and Fisher exact test were used for comparisons between proportions. STARD guidelines were followed for reporting and analyses (43). Analyses were generated using Stata 14.0 special edition software (Stata statistical software, release 14; StataCorp LP, College Station, TX).

This study was approved by the Stellenbosch University Health Research Ethics Committee (reference N11/09/282) and by local health authorities.
